# Case report: Granzyme-B expression by T- and B- cells during severe AQP4-positive Neuromyelitis Optica spectrum disorder with fatal venous thromboembolism outcome

**DOI:** 10.3389/fneur.2023.1208977

**Published:** 2023-08-17

**Authors:** Vinícius Oliveira Boldrini, Mariana Rabelo Brito, Raphael Patrício Silva Quintiliano, Lucas Scárdua Silva, Clarissa Lin Yasuda, Fernando Cendes, Alessandro Santos Farias, Alfredo Damasceno

**Affiliations:** ^1^Neuroimaging Laboratory, Department of Neurology, University of Campinas, Campinas, São Paulo, Brazil; ^2^Brazilian Institute of Neuroscience and Neurotechnology (BRAINN), University of Campinas, Campinas, São Paulo, Brazil; ^3^Neuroimmunology Unit, Department of Genetics, Evolution, Microbiology and Immunology, Institute of Biology, University of Campinas, Campinas, São Paulo, Brazil; ^4^Autoimmune Research Laboratory, Department of Genetics, Evolution, Microbiology and Immunology, Institute of Biology, University of Campinas, Campinas, São Paulo, Brazil

**Keywords:** Devic's syndrome, deep vein thrombosis (DVT), pulmonary thromboembolism, T lymphocytes, B cells, granzyme-B, neuroinflammation, anti CD20 monoclonal antibody

## Abstract

**Background:**

The expression of serine protease granzyme-B (GzmB) by circulating CD8^+^ T lymphocytes has been recently suggested as a biomarker for poor immunotherapy response and severe disability in patients with Neuromyelitis Optica spectrum disorders (NMOSD). In parallel, venous thromboembolism (VTE) has been reported mainly in NMOSD patients exhibiting transverse myelitis.

**Case presentation:**

Here, we describe an Aquaporin-4 positive (AQP4-positive) NMOSD patient who showed short myelitis (SM) and experienced a fatal pulmonary thromboembolism/lower extremity deep vein thrombosis during anti-CD20 treatment. Flow cytometry analyses from the peripheral blood revealed an enhanced cytotoxic behavior through circulating CD8^+^GzmB^+^ T, CD4^+^GzmB^+^ T lymphocytes, and residual CD19^+^GzmB^+^ B cells.

**Conclusions:**

Fatal VTE may be a rare outcome, particularly in patients exhibiting SM, and may share poorly understood immunological mechanisms with AQP4-positive NMOSD severity.

## Background

Neuromyelitis Optica spectrum disorder (NMOSD) is an autoimmune neuro-inflammatory disease that affects the central nervous system (CNS) with a propensity for causing lesions in the optic nerves and spinal cord. The discovery of autoantibodies such as specific NMO-IgG against Aquaporin-4 (AQP4-IgG) (1) and the clinical efficacy of anti-CD20 therapies highlight the importance of humoral factors related to B cells during disease (2). On the other hand, recent reports emphasize the role of serine-protease granzyme-B (GzmB) as an effector mechanism derived from CD8^+^ T lymphocytes during NMOSD (3, 4). This cytotoxic behavior of CD8^+^ T lymphocytes, which in some resembles well-known mechanisms of immunopathology from Multiple Sclerosis (MS) (5–11), was suggested as a potential biomarker for clinical severity and poor immunotherapy response in NMOSD individuals (4).

Considering it, here we describe a patient who exhibited enhanced GzmB expression by circulating T- and B- subsets during rituximab therapy and further developed fulminant venous thromboembolism (VTE) in a severe course of AQP4-positive NMOSD.

## Case presentation

**Figure 1:**
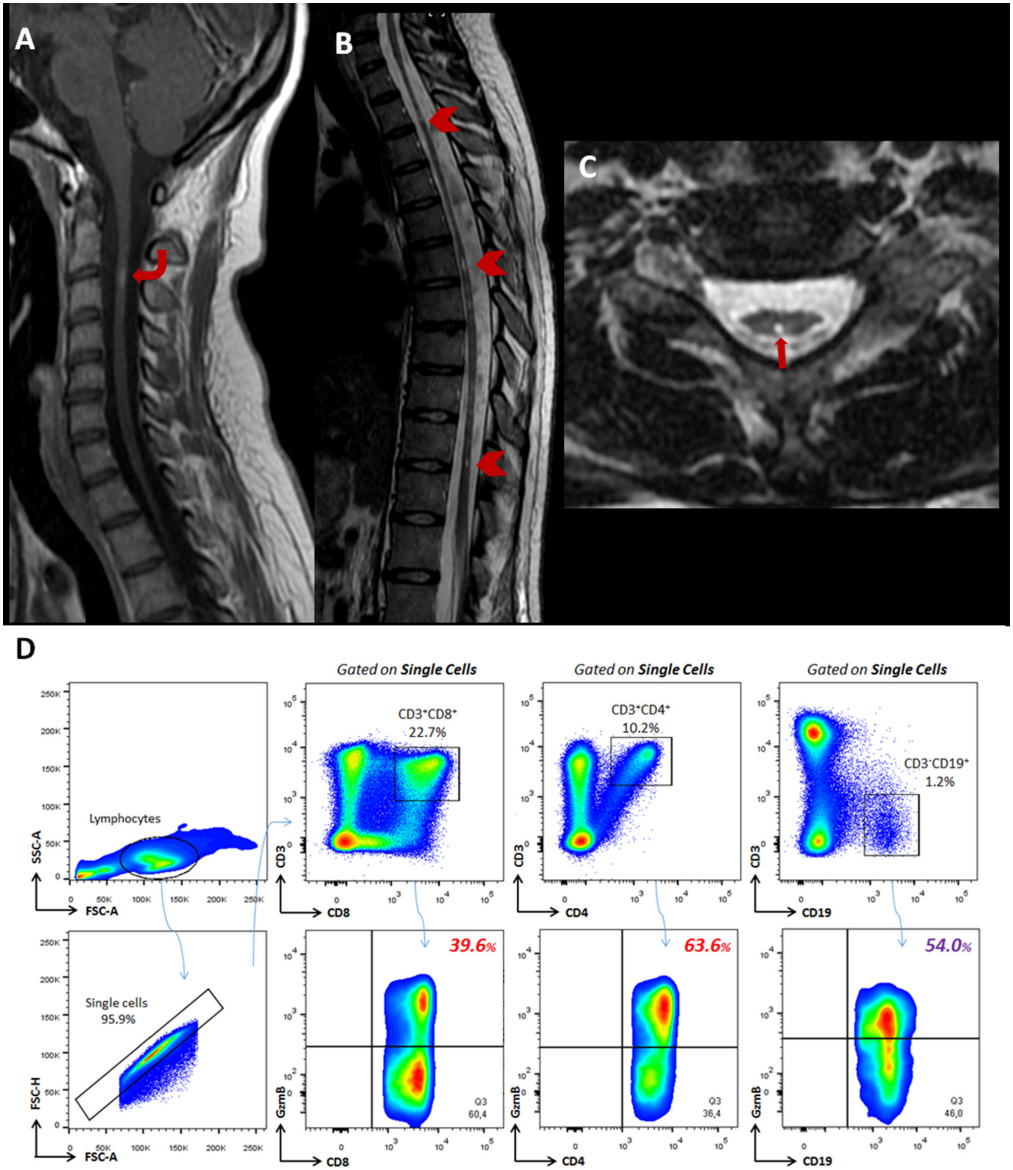
Imaging and profile of cytotoxic T- and B- subsets from a patient with AQP4-positive NMOSD. MR images of the cervical and thoracic spine demonstrate spinal cord involvement. **(A)** A sagittal contrast-enhanced T1-weighted image of the cervical spine shows a focal enhancing lesion at the C2-3 level (curved arrow). **(B)** The sagittal T2-weighted image shows diffuse thoracolumbar spinal cord atrophy and some multiple short segments of hyperintensity (arrowhead) along the spinal cord. This pattern may be present in chronic phases, and lesions tend to fragment into shorter segments after high-dose steroids or during remission. **(C)** The axial T2-weighted image shows a bright spotty lesion located centrally at the T2 level (arrow) and also demonstrates spine cord atrophy. **(D)** Flow cytometry analyses show percentages of CD8^+^GzmB^+^ (39.6%) and CD4^+^GzmB^+^ (63.6%) T lymphocytes beyond CD19^+^GzmB^+^ B cells (54.0%) identified in the peripheral blood.

**Figure 2:**
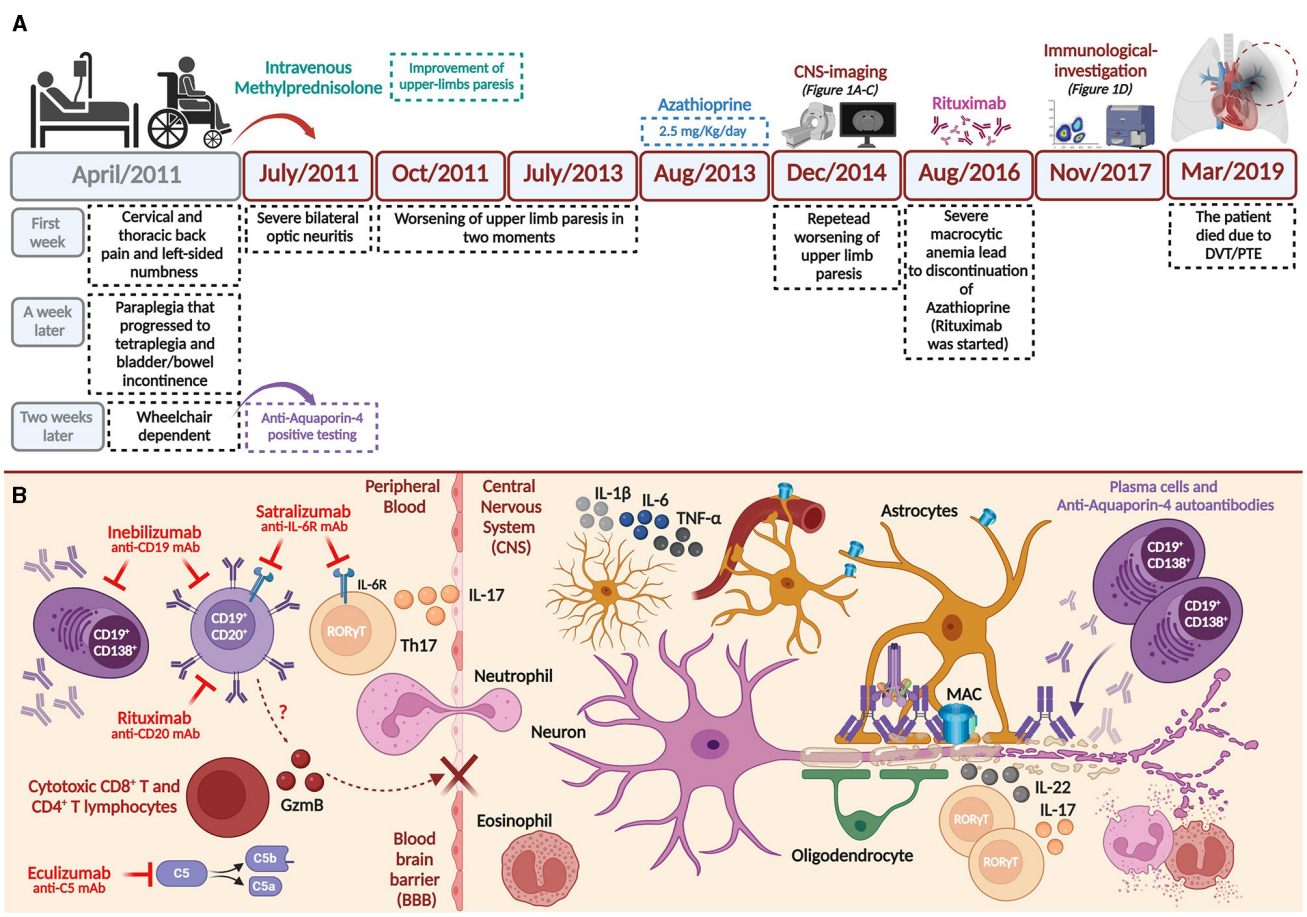
Clinical history and hypothesis on the involvement of cytotoxic T- and B- subsets during AQP4-positive NMOSD physiopathology. **(A)** Main findings of the clinical history until deep vein thrombosis (DVT)/pulmonary thromboembolism (PTE) outcome. **(B)** Th17 subsets (expressing IL-17), innate cells, and complement system may promote peripheral inflammation, eventually disrupting the blood-brain barrier (BBB) during AQP4-positive NMOSD pathogenesis (2, 30, 31). Considering well-established pathophysiological mechanisms during disease, approved monoclonal antibodies used to treat NMOSD are shown (28). We hypothesize that cytotoxic (CD8^+^ and CD4^+^) T- and B- subsets may also support BBB damage by expressing and releasing serine-protease granzyme-B (GzmB). Thus, once AQP4-IgG antibodies enter the central nervous system (CNS), they bind with astrocyte end-feet characterizing a primarily astrocytopathy. Deposition of complement factors is also observed (C1q, among other factors) and terminally leads to the formation of membrane attack complex (MAC). Reactive astrocytes can release pro-inflammatory cytokines (e.g., IL-1, IL-6, TNF-α) upon stimulation. Eosinophils, neutrophils, and other innate cells (e.g., macrophages, *not shown*) are also implicated in the oligodendrocyte and neuronal loss secondarily to the astrocytopathy (2, 30, 31). Created with Biorender.com.

A woman in her early 20s presented initially with cervical and thoracic back pain and left-sided numbness, during the second trimester of pregnancy, in April/2011. A week later, she had paraplegia that progressed to tetraplegia and bladder/bowel incontinence. At the nadir, 2 weeks after onset, she was wheelchair dependent. A cerebrospinal fluid (CSF) study revealed 10 cells/mm3 (49.5% lymphocytes, 47.5% neutrophils, 2% monocytes, and 1% eosinophils), protein of 42 mg/dL (normal, 0 to 45 mg/dL), and negative oligoclonal bands (OCBs). Serum cell-based assay AQP4-IgG was positive. She was treated with a 5-day course of intravenous methylprednisolone (IVMP) 1 g daily with some improvement in her upper limbs paresis. In July/2011, the patient had a severe bilateral optic neuritis relapse. In October/2011, she had worsening upper limbs' paresis, which repeated on July/13 and December/14, reaching an expanded disability status scale (EDSS) of 8.0. Cervical spine MRI showed a focal enhancing lesion at the C2-3 level and spinal cord atrophy (Figures 1A–C). In August/2013, she started to attend our outpatient clinic and was diagnosed with NMOSD, and commenced azathioprine (2.5 mg/kg/d) until August/2016, when she presented severe macrocytic anemia. At this time, the treatment was switched to the chimeric anti-CD20 monoclonal antibody (mAb) (rituximab). In November/2017, she presented a persistent EDSS of 8.0 and agreed to participate in our study. Similarly to the protocol mentioned (10), a blood sample was collected, and peripheral blood mononuclear cells (PBMCs) were obtained to investigate cytotoxic-related functions in circulating T- and B- cells using flow cytometry analyses. In March/2019, the patient died due to pulmonary thromboembolism (PTE) and lower extremity deep vein thrombosis (DVT) (clinical history is summarized in Figure 2A).

## Discussion

To our knowledge, we show here, for the first time, a fatal case of VTE in an AQP4-positive NMOSD patient exhibiting short myelitis (SM) during rituximab treatment. The diagnosis of NMOSD itself seems to increase the odds of VTE compared to MS patients (12). Recently, VTE was suggested in 12.0% of total NMOSD attacks (13). Among NMOSD phenotypes, patients presenting transverse myelitis (TM) were more prone to develop VTE. Age of attacks, acute infections/or rheumatologic disorders, and intravenous immunoglobulin (IVIG) treatment were significantly associated with VTE in the subgroup of NMOSD patients exhibiting TM (13). Besides it, VTE was also reported during cerebral syndrome, simultaneous TM and brainstem syndrome, but no occurrence of VTE was observed so far in patients exhibiting SM (13).

In our case, about 1.3 years before the patient developed fatal VTE, we found enhanced cytotoxic behavior from classical CD8^+^GzmB^+^ T (39.6%), non-classical CD4^+^GzmB^+^ T (63.6%) and CD19^+^GzmB^+^ B (54.0%) lymphocytes during chimeric anti-CD20 mAb (Figure 1D). Similarly to our present data, Shi and coworkers found higher circulating CD8^+^GzmB^+^ T lymphocytes in NMOSD patients (50.2%) compared to healthy donors (27.1%). Their analysis indicated a cut-off value of 49.6% of circulating CD8^+^GzmB^+^ T lymphocytes for poor response to immunotherapy (azathioprine/mofetil mycophenolate and rituximab). In poor responders, CD8^+^GzmB^+^ T lymphocytes positively correlated with EDSS scores and serum levels of neurofilament light chain (NfL) and glial fibrillary acidic protein (GFAP) (4). Of note, we have previously described a massive percentage of CD8^+^GzmB^+^ T and CD4^+^GzmB^+^ T subsets, in the CSF and peripheral blood, from an AQP4-positive NMOSD patient who was resistant to the acute treatment for relapses (14).

Except for these previously mentioned studies (4, 14), until now, further investigations on CD8^+^GzmB^+^ T lymphocytes and other non-classical cytotoxic subsets have yet to be further performed in NMOSD patients. On the other hand, and particularly during MS (5–11), increasing evidence supports the involvement of GzmB-derived from T lymphocytes during neuro-inflammatory conditions (15–19). In MS patients, classical CD8^+^ T and non-classical (such as CD4^+^ T) lymphocytes have been related to disease activity and prognosis (6, 11, 20).

In our Brazilian cohort, relapsing-remitting MS (RRMS) patients show about 34.0% CD8^+^GzmB^+^ T- and 13.0% CD19^+^GzmB^+^ B- lymphocytes, while healthy donors show about 20.0% and 2.0%, respectively, from these circulating subsets (10). Moreover, GzmB expression was significantly higher in CD57^+^ (*vs*. CD57^−^) and CD28^null^ (*vs*. CD28^+^) subsets of CD8^+^ T lymphocytes in MS patients (10). Indeed, other reports indicate that CD8^+^CD28^null^CD57^+^ T lymphocytes exhibit enhanced cytotoxic activity even higher than NK cells, as well as may exhibit reactivity against Epstein-Barr virus (EBV) (6) and autoantigens, such as myelin (21). CD8^+^CD57^+^ T lymphocytes are infiltrated in meninges and found in MS post-mortem tissue. When this subset highly expresses immune-checkpoint programmed death-1 (PD-1), it exhibits impaired cytotoxic behavior (6) and defective control of EBV replication associated with MS relapses. Notably, massive GzmB-derived from EBV-reactive T lymphocytes were described in the CNS parenchyma from fulminant MS relapses after anti-CD49d mAb discontinuation (7, 8, 22).

Interestingly, regarding treatment, besides the release of GzmB is thought to be mainly derived from NK cells due to antibody-dependent cellular cytotoxicity (ADCC) during anti-CD20 mAb treatment (23), there is also evidence obtained in cancer patients supporting at least a transient effect of rituximab on cytotoxic CD8^+^GzmB^+^ T lymphocytes (24, 25). The precise mechanism of action for it still needs to be better understood.

These findings reinforce the importance of further investigations on soluble factors such as GzmB during anti-CD20 mAb treatments.

A limitation of our report is that we could not measure GzmB expression by circulating T- and B- subsets in two other important time points, such as before the initiation of rituximab and near the moment of the patient's death. However, resembling data from MS (6, 11, 20) and recent evidence obtained in NMOSD (4), it is possible to speculate that arising/continuous cytotoxic T- and B- subsets may impact the clinical severity (due to constant EDSS 8.0) and poor response to rituximab observed in our patient. Mechanistically, GzmB is known to cause damage to the blood-brain barrier (26), possibly allowing the entry of inflammatory subsets and AQP4-IgG into the CNS during NMOSD (27, 28) (summarized in Figure 2B).

Particularly considering B cells, further investigation may address whether residual non-classical CD19^+^GzmB^+^ B cells may sustain antibody-independent functions in AQP4-positive NMOSD patients under anti-CD20 mAb treatment. By now, it's possible to hypothesize that recently approved inebilizumab (29), anti-CD19 mAb for NMOSD, would promote a more efficacious depletion of CD19^+^GzmB^+^ B cells than anti-CD20 therapies since these subsets apparently lack the expression of CD20 (10). Curiously, anti-CD19 therapy has been suggested to escalate after anti-CD20 use in unresponsive NMOSD patients (29).

## Data availability statement

The original contributions presented in the study are included in the article/supplementary material, further inquiries can be directed to the corresponding authors.

## Ethics statement

The studies involving human participants were reviewed and approved by the University of Campinas Committee for Ethical Research, according to the Term of Consent (CAAE: 53022516.3.0000.5404). The patients/participants provided their written informed consent to participate in this study. Written informed consent was obtained from the individual(s) for the publication of any potentially identifiable images or data included in this article.

## Author contributions

VB and RQ performed flow cytometry experiments. MB, LS, and AD selected the patient, proceeded and analyzed the MRI, as well as wrote clinical data. VB, CY, FC, AF, and AD designed and coordinated the study. VB, MR, and AD wrote the manuscript with inputs from co-authors. All authors contributed to the article and approved the submitted version.
